# Self-Serving Dishonesty Partially Substitutes Fairness in Motivating Cooperation When People Are Treated Fairly

**DOI:** 10.3390/ijerph19106326

**Published:** 2022-05-23

**Authors:** Dandan Li, Ofir Turel, Shuyue Zhang, Qinghua He

**Affiliations:** 1 MOE Key Laboratory of Cognition and Personality, Faculty of Psychology, Southwest University, Chongqing 400715, China; 18260079751@163.com; 2 Computing Information Systems, The University of Melbourne, Parkville, VIC 3010, Australia; oturel@exchange.fullerton.edu; 3 Guangxi Colleges and Universities Key Laboratory of Cognitive Neuroscience and Applied Psychology, Faculty of Education, Guangxi Normal University, Guilin 541006, China; 4 Collaborative Innovation Center of Assessment toward Basic Education Quality at Beijing Normal University, Southwest University Branch, Chongqing 400715, China

**Keywords:** fairness, cooperation, honesty, the prisoner’s dilemma, die-rolling task

## Abstract

Fairness is a key expectation in social interactions. Its violation leads to adverse reactions, including non-cooperation and dishonesty. The present study aimed to examine how (1) fair (unfair) treatment may drive cooperation (defection) and honesty (self-serving dishonesty), (2) dishonesty primes further moral disengagement and reduced cooperation, and (3) dishonesty weakens (substitutes) the effect of fairness on cooperation. The prisoner’s dilemma (Experiment 1 and 2) and die-rolling task (Experiment 2) were employed for capturing cooperation and dishonest behaviors, respectively. To manipulate perceived unfairness, participants were randomly assigned to play the prisoner’s dilemma game, where players either choose more cooperation (fair condition) or defection (unfair condition). Results of Experiment 1 (*n* = 102) suggested that participants perceive higher unfairness and behave less cooperatively when the other player primarily chooses defection. Results of Exp. 2 (*n* = 240) (a) confirmed Exp. 1 results, (b) showed that players in the unfair condition also show more self-serving dishonest behavior, and (c) that dishonest behavior weakens the effect of fairness on cooperation. Together, these results extended previous work by highlighting the self-serving lies when the opponent is fair trigger higher cooperation, presumably as a means to alleviate self-reflective moral emotions or restore justice.

## 1. Introduction

Fairness has been a key principle in social interactions since the dawn of humanity, as it dictates social expectations and defines acceptable social behaviors [1]. As such, fairness norms play a crucial role in social interactions across cultures [2]. Toddlers as young as 2 years old learn to perceive the fairness of social interactions and develop a preference for fair interactions, and this preference further develops as they grow [3]. As such, most humans judge social interaction, at least in part, based on their fairness, by answering the questions “was this action reasonable, right, and just?” [4], and fairness perception influences a wide range of social and professional interactions [5]. Importantly, violations of fairness norms and expectations (perceived unfairness) can lead to adverse emotional and behavioral responses [6,7,8,9,10,11,12], and these can negatively affect social interactions and their outcomes [5,13,14,15].

Given the broad effects of fairness, it has been studied from various theoretical angles. For example, in economics, fairness refers to a socially acceptable distribution of money or other goods [4]; in organizations, it refers to socially acceptable treatment of employees [9]. Importantly, in all of the theoretical treatments of fairness, it has been modeled as reciprocal. Fair behaviors are reinforced by reciprocally fair behaviors, whereas hostile or unfair acts are reciprocated with hostility [16] and inequity aversion efforts [17,18,19,20]. This happens because when people are treated unfairly, they trade-off fairness goals against the goal of increasing their material resources as a means to restore the sense of fairness [21].

Here, we focus on cooperation as an outcome of sensing fairness (or unfairness), because fairness is a key basis upon which people decide to cooperate, and cooperation is an important mechanism that underlies many social and business processes. When cooperation involves financial decisions, fairness reflects “the concern for how money or goods are distributed among individuals” [22]. Possible effects of fairness on cooperation can be explained through inequity aversion theory, according to which people compare their payoffs with those of others and prefer payoffs that are more equally distributed [23,24,25]. Bolton and Ockenfels as well as Fehr and Schmidt suggested that the desire to adjust payoff differences among people reflects their fairness concerns. Indeed, inequality in division of resources motivates attempts to restore equity, even via risky behaviors [26].

Another common response to fairness (unfairness) is honest (dishonest) behavior. This association is based on equity theory, according to which people try to restore equity when treated unfairly, in part through deception, unethical, and dishonest behavior [27,28]. This idea is also consistent with the Broken Window Theory, which posits that people are less likely to adhere to social norms in environments where violation of social norms is easily noticeable [29]. Therefore, experiencing unfairness can increase dishonesty [30]. Laboratory studies [30,31,32] have supported such assertions.

Our study, therefore, aims to respond to prior studies’ calls for investigating whether individuals’ inclination to cooperate is fairness at first [33]. Although, there are lots of studies that have investigated the relationship between prosocial behavior and lying aversion and found that the more prosocial behavior, the less lying because of the lying aversion [34,35,36,37]. The subjects of these studies play a two-stage game; in the first stage, they are assigned to play either the DG (Dictator game) or the PD (Prisoner’s Dilemma game); in the second stage, they are assigned to a Deception Game [34,38]. Our study combined the PD and lying paradigm and aimed at integrating the streams of research on cooperation and dishonesty in response to unfair treatment.

Considering the research literature, we conducted an incentivized study investigating the research questions and hypotheses described below. Firstly, taking into consideration the scarcity of studies demonstrating the impact of fairness on cooperation and dishonesty, we aimed to verify previous findings that perceived fairness affects cooperation and dishonesty. Therefore, we hypothesized the following:

**Hypothesis** **1** **(H1).** *Participants will cooperate less and be more dishonest in the unfair treatment condition (when they perceive to be treated unfairly).*

Secondly, based on the literature and the reasoning presented in the previous paragraph, we formulated a research question concerning the relation between fairness, cooperation, and dishonesty.

On the one hand, lying can serve as a basis for reduced cooperation, and further deteriorate social interaction. This may be explained by moral disengagement theories [39,40], according to which unethical behaviors in one domain can trivialize and encourage unethical behaviors in another, and help people morally disengage and perform such behaviors. It can also be explained from a social exchange/equity restoration perspective, according to which people can lie, deceive, and steal to restore their sense of justice [41]. As such, lying on one task may further prime people to reduce reliance on moral norms, and beyond the unfair treatment effect, motivate justice restoration through focusing on one’s own needs, contributing to lower cooperation.

On the other hand, lying can serve as a means to restore equity in social exchange in response to unfair treatment [42]. It may therefore cannibalize from the motivation to defect (another means to restore equality) as created by unfair treatment; people may feel a lesser need to respond in defection to unfair treatment if they are already lying. In this sense, lying and non-cooperation can serve as substitute means to restore fairness. Furthermore, when people lie even though they were treated fairly, they are expected to regret and feel ashamed and/or guilty about their actions [43]. Therefore, they may feel compelled to alleviate such unpleasant moral emotions through corrective action [44,45,46,47], plausibly in the form of increased cooperation. That is, in addition to direct effects of unfair treatment on lying and defection, we expect lying to weaken the effect of fairness on cooperation, and even serve to increase cooperation when people are treated fairly (a case that will likely invoke the highest expected negative moral emotions).

**Hypothesis** **2** **(H2).** *Dishonesty will partially mediate the effect of unfairness on cooperation, dishonesty will weaken the effect of unfairness on cooperation, and this effect will be pronounced when people are treated fairly, and vice versa*.

In this study, we manipulated fairness perceptions by employing the prisoner’s dilemma (PD) game in Experiments 1 and 2, in which two agents can either cooperate (c) or defect (d): cooperating means paying a cost c to give a benefit b (b > c) to the other person; defecting means doing nothing [48]. In the PD, if both players defect, both are worse off than if both cooperate. Despite this, players often choose defection over cooperation [49]. We measured dishonesty with a die-rolling task in the experiment 2. This paradigm overcomes some reporting biases [32,50]. Using this paradigm, studies have shown that people who perceive unfairness tend to misreport the outcome in a self-serving direction in the coin flipping game [30,51] and in the die-rolling task [52,53,54]. Experiment 1 aimed to test if people reduce their cooperation levels when they are treated unfairly, as compared to under fair treatment. Experiment 2 used both the PD and the die-rolling tasks to test whether the perceived unfairness primed by opponents’ choices affected cooperation and honesty. We specifically incentivized self-serving dishonesty with three mechanisms: (a) adding the die rolling points to one’s own account, or (b) adding to the opponent’s account, or (c) deducting the points from the opponent’s account.

## 2. Experiment 1

Experiment 1 aimed to test if people will cooperate less when they are subjected to perceived unfairness (i.e., when they are in the unfair condition as compared to the fair condition). We employed a two-person prisoner’s dilemma game in which participants were instructed to play with pre-assigned players whom they saw, and which were then replaced (after the player stepped into a separate room) by preprogramed algorithms (i.e., players thought they are playing against a person). Participants were randomly assigned to play this game with either a fair player (whose choices were 80% cooperation) or an unfair player (whose choices were 80% defection). Participants were instructed to choose to cooperate or defect in each of the 50 rounds they played. The payoff matrix of the game is shown in the following table (Table 1).

### 2.1. Materials and Methods

#### 2.1.1. Participants

We used G*Power 3.1 to determine a priori that at least 90 participants would be needed to detect a larger effect (Cohen’s *d* = 0.6) with a probability of 0.80 (1−β) [55]. The sample included 102 students (*M* = 19.10 years old, *SD* = 1.38; 84 females) who were voluntarily recruited from a university in Chongqing province in China via the Internet. All participants were right-handed with normal or corrected vision and without neurological or psychiatric history. The participants received compensation (about 20~30 RMB) at the end of the study. Written informed consent, which was approved by local Institutional Review Board, was obtained from all participants. The full sample was collected prior to running any analyses, and no participants were excluded from the data analysis.

#### 2.1.2. Study Design and Procedure

The experiment employed a simple between-subject design (fair vs. unfair) with the prisoner’s dilemma task.

The prisoner’s dilemma task was presented using e-prime (version 2.0, Psychology Software Tools Inc., Sharpsburg, PA, USA). After reporting basic demographic data, participants were informed that they were going to play multiple rounds of a two-person prisoner’s dilemma game with a randomly matched player (a confederate), who was introduced to them.

**Figure 1:**
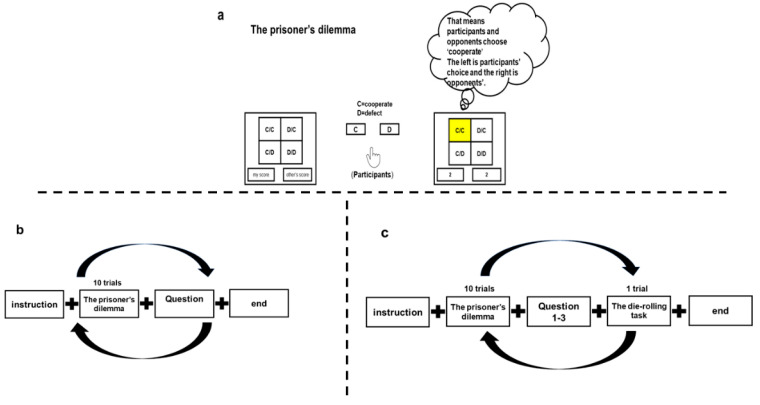
(**a**) The prisoner’s dilemma paradigms: Examples of an interaction sequence with the playing partner (right icon). After instruction, the participant had to decide between the defective (D) and the cooperative (C) choice, which was followed by the presentation of buttons (my score and other’s score) depicting the choice and outcome for both players. The left side (in color) is the participant’s choice and the right side is the opponent’s choice. The payoff started by zero at the start and then was accumulated. (**b**) Experiment flow chart. At the beginning, participants chose to “cooperate” or “defect” with opponents (confederates that participants met who were replaced by computer algorithms). After each block of 10 rounds, participants were asked to respond on 1–7 scales to a question about perceived fairness rating: “In the previous block, do you think the opponent’s choices were fair to you (1 = very unfair, 7 = very fair)?” the experiment had 60 rounds/trials. (**c**) Experiment flow chart. At the beginning, participants chose to “cooperate” or “defect” with opponents (who were actually confederates who were replaced by computer algorithms). After each block of 10 rounds, (1) participants were asked to respond on1–7 scales to three questions: (1) On average, how was your counterpart in this block of trials (1 = not at all cooperative, 7 = very cooperative)? (2) In the next block of trials, do you expect that your counterpart will cooperate or betray more (1 = betray more, 7 = cooperate more)? and (3) In the previous block, do you think the opponent’s choices were fair to you (1 = very unfair, 7 = very fair)? Questions 1 and 2 checked whether participants understand the experiment and behaved rationally. Question 3 captured perceptions of the fairness of the opponent, to secretly roll a die, and to report the obtained points. The study had 60 trials of prisoner’s dilemma and 10 trials of die-rolling task.

Participants were asked to complete 60 trials of the game, organized in five 10-game blocks. After each block of 10 trials, participants were asked to respond to the following question: “In the previous block, do you think the opponent’s choices were fair to you (1 = very unfair, 7 = very fair)?” This question captured perceptions of the fairness of the opponent. Participants started the game after comprehension checks and three practice sessions. The flow of the experiment is depicted in Figure 1.

### 2.2. Results

**Figure 2:**
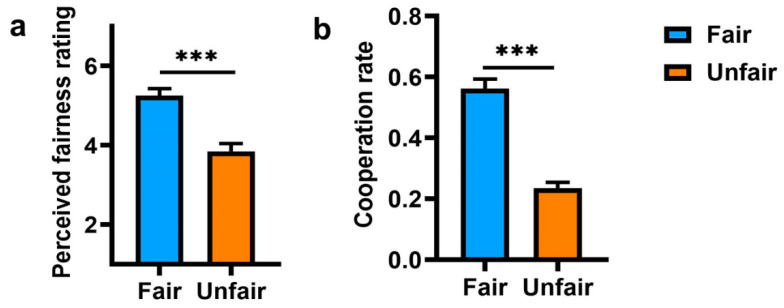
Results from Exp. 1. (**a**) Mean perceived fairness rating in fair and unfair conditions; (**b**) mean cooperation in fair and unfair conditions, presented separately for participants randomly assigned to players whose choices were either 80% cooperation or 80% defection. Error bars indicate standard error. ***: *p* < 0.001.

First, we examined the perceived fairness in the different condition by employing an independent sample *t*-test. The results showed that participants reported higher (*t*(100) = 5.15, *p* < 0.001, Cohen’s *d* = 1.02, 95% CI = [0.51, 1.53]) perceived opponent fairness in the cooperation/fair condition (*M* = 5.25, *SD* = 1.29) than in the defection/unfair condition *(M* = 3.85, *SD* = 1.46) (Figure 2a). This suggested that the participants had normal fairness perceptions, which differed in the expected direction.

Further, cooperation rates in 50 trials were averaged and submitted to an independent sample t-test. As predicted (Figure 2b), there was a significant difference between treatments (fair vs. unfair), *t*(100) = 9.11, *p* < 0.0001, Cohen’s *d* = 1.80, 95% CI = [1.34, 2.26], such that participants’ cooperation rate was higher in the fair condition (*M* = 0.56, *SD* = 0.22) than in the unfair condition (*M* = 0.24, *SD* = 0.13).

## 3. Experiment 2

Experiment 2 aimed to replicate and extend the results from Experiment 1. Extensions included measuring dishonesty. Dishonesty was measured using the die-rolling task, in which each participant throws a die privately—so that only he or she can see the number thrown—and reports the outcome for which the participant gets paid depending on the reported number [32,56]. Because the die roll is strictly private, participants can dishonestly report a higher number than they threw. Lying can be detected from significant deviations from the probability of each number being thrown (i.e., 16.7%) [32,57]. To this end, after each block of 10 trials, participants (1) completed a three item seven-point Likert-scale that captured manipulation checks (experience with the opponent in the previous block and expectations for the next block) and perceptions of fairness, and (2) reported points on a die rolling task, which was used for measuring dishonesty in the different conditions. Participants were told that the number they reported would be used to either add the same amount to their tally or deduct the same amount from their opponent’s tally.

This was done to examine how fairness and lying can produce different cooperation levels, when different point allocation systems are employed. For comprehension check purposes, we expected that if participants comprehended the game, their experience with the opponent in the previous block would inform their expectations for the next block; and that these experiences and expectations would inform their rate of cooperation. We also expected that people in the fair condition would be more honest than those in the unfair condition.

### 3.1. Materials and Methods

#### 3.1.1. Participants

We used G*Power 3.1 to determine a priori that at least 149 participants would be needed to detect a larger effect (f = 0.4) with a probability of 0.80 (1−β) [55]. A total of 240 undergraduate students from a university campus (189 females, *M* = 19.30, *SD* = 1.20) were voluntarily recruited from a university in Chongqing province in China via the Internet. Participants received compensation of 20–30 RMB at the end of the study. Written informed consent, which was approved by local Institutional Review Board, was obtained from all participants. The full sample was collected prior to running any analyses, and no participants were excluded from the data analysis.

#### 3.1.2. Study Design and Procedure

The experiment followed a between-subjects design, which consisted of two fairness conditions (fair vs. unfair) and three-point systems (added to self vs. deducted to others vs. added to others).

1. As in Experiment 1, the proportion of “cooperation” choices in the prisoner’s dilemma game served as the index for cooperative behavior.

2. The distance of the average number of points reported by the participants from the expected value (3.5) served as a measure of lying. If all participants reported the true number, all numbers would have a similar probability of 1/6, and the mean reported points (expected value) is 3.5. This served as the honesty benchmark. Hence, reported average die-rolling points greater than 3.5 were considered as self-serving lying in cases where points were added to participants’ accounts or deducted from opponents’ accounts. Reported average die-rolling points less than 3.5 were considered as self-serving lying in cases were the obtained points were added to the opponent’s account.

After this classification, cooperation rates were subjected to a 2 (fairness condition: fair vs. unfair) × 3 (point system: added to self vs. added to opponents vs. deducted from opponents) between-subjects ANOVA.

E-prime (version 2.0, Psychology Software Tools Inc., Sharpsburg, PA, USA) was used to present the stimuli and collect the responses. Before the experiment, participants were briefly introduced to their opponents who were confederates of the experimenter. They were not allowed to communicate with each other. Then, participants and opponents went to different soundproof rooms (the confederates did not actually play; an algorithm did). Next, participants watched the experimenter trying to connect with the player he/she just met to make it realistic. Participants were asked to complete 60 trials of the game, organized in six 10-game blocks.

After each block of 10 trials, they were asked to respond on a 1–7 scale to the following three questions: (1) On average, how was your counterpart in this block of trials (1 = not at all cooperative, 7 = very cooperative)? (2) In the next block of trials, do you expect that your counterpart will cooperate or betray more (1 = betray more, 7 = cooperate more)? and (3) In the previous block, do you think the opponent’s choices were fair to you (1 = very unfair, 7 = very fair)? Questions 1 and 2 checked whether participants understood the experiment and behaved rationally. Question 3 captured perceptions of the fairness of the opponent. At the end of each block of 10 trials, participants were also asked to secretly throw a die, and then report the obtained points. They knew that the reported points would be either added to their account, added to their opponent’s account, or deducted from their opponent’s account. The flow chart of the experiment is depicted in Figure 1a,c.

### 3.2. Results

Participants’ reported cooperation levels of the opponent in the previous block, as well as their cooperation expectations for the next block, were averaged across blocks. The average cooperation experience and expectation scores were highly correlated (r = 0.88, *p* < 0.001), and the cooperation experience and expectation scores were highly correlated with the average cooperation rate (r = 0.59, r = 0.51, *p* < 0.001). In addition, participants reported higher (*t*(238) = 9.24, *p* < 0.001, Cohen’s *d* = 1.19, 95% CI = [0.92, 1.47]) perceived opponent fairness in the cooperation/fair condition (*M* = 5.27, *SD* = 0.98) than in the defection/unfair condition (*M* = 3.69, *SD* = 1.60). This suggested that the participants understood the game, developed normal behavior-contingent expectations, and had normal fairness perceptions, which differed in the expected direction.

#### 3.2.1. Cooperation Rate

**Figure 3:**
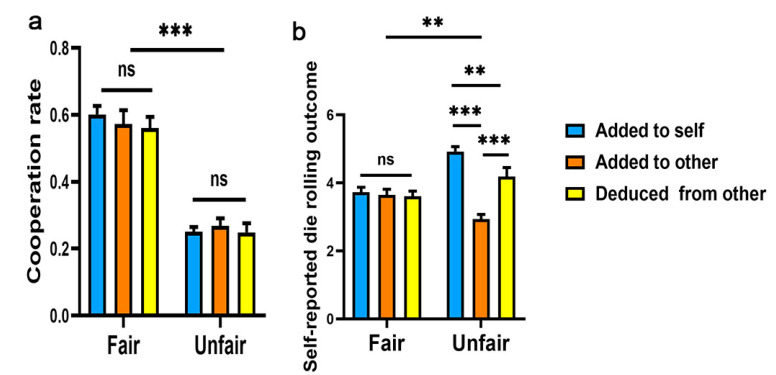
Results from Exp. 2. Averaged (**a**) cooperation rate and (**b**) outcome of die-rolling as a function of point system (added to self vs. added to opponent vs. deducted from opponent), separately for the fair condition and the unfair condition. Error bars indicate standard error. **: *p* < 0.01,***: *p* < 0.001, ns: no significant difference.

Group means of cooperation and lying, by condition, are presented in Figure 3a. We tested the significance of cooperation differences with a 2 (fairness condition: fair vs. unfair) × 3 (point system: added to self vs. added to opponents vs. deducted from opponents) between-subjects ANOVA. Results first replicated the finding of Exp. 1 with a significant effect of fairness condition (*F*(1, 234) = 198.56, *p* < 0.001, *η_p_²* = 0.46, 95% CI = [0.37, 0.53]), suggesting that people cooperated significantly more in the fair condition (*M* = 0.58, *SD* = 0.20) than in the unfair condition (*M* = 0.25, *SD* = 0.13). There was no significant main effect of point allocation system (*F*(1, 234) = 0.34, *p* = 0.71), or fairness condition × point system interaction (*F*(1, 234) = 0.5, *p* = 0.61).

#### 3.2.2. Degree of Lying

Next, we examined differences between the groups in terms of the points participants reported in the secret die-rolling task as an index of (dis)honesty, with a 2 (fairness condition: fair vs. unfair) × 3 (point system type: added to self vs. added to opponent vs. deducted from opponent) between-subjects ANOVA, as presented in Figure 3b. There was a main effect of fairness condition (*F*(1, 234) = 5.79, *p* = 0.017, *η_p_*² = 0.02, 95% CI = [0.001, 0.08]), suggesting that people lied more in the unfair condition (*M* = 4.24, *SD* = 1.40) than in the fair condition (*M* = 3.68, *SD* = 1.02). The reported points in fair conditions is not significantly different from 3.5 (*t*(119) = 1.88, *p* = 0.062, Cohen’s *d* = 0.18, 95% CI = [−0.01, 0.36]) for the fair condition; however, the reported points in unfair condition is significantly higher than 3.5 (*t*(119) = 4.30, *p* < 0.001, Cohen’s *d* = 0.39, 95% CI *=* [0.21, 0.58]), suggesting that participants lied more in unfair condition to self-serve their interests.

There was also a main effect of point system (*F*(1, 234) = 18.25, *p* < 0.001, *η_p_*² = 0.14, 95% CI = [0.02, 0.14]), indicating that participants reported more points when points were added to their accounts (*M* = 4.32, *SD* = 1.28) compared to when points were deducted from the opponent’s account (*M* = 3.89, *SD* = 1.22) or when points were added to the opponent’s account (*M* = 3.29, *SD* = 0.91). There was a significant fairness condition × point system interaction. (*F*(1, 234) = 15.41, *p* < 0.001, *η_p_*² = 0.12, 95% CI = [0.02, 0.13]). Simple effect tests showed a significant effect in the unfair condition (*F*(2, 234) = 33.51, *p* < 0.001, *η_p_²* = 0.22, 95% CI = [0.14, 0.31]).

#### 3.2.3. The Relation between Fairness, Cooperation, and Lying

To examine whether lying moderates the effect of fairness on cooperation, two moderation analyses were conducted: one with a between-subjects ANOVA treating lying as a binary variable, and another with regression analysis, treating lying as a continuous concept, where lying is the distance from the expected value (3.5). In addition, self-reported points over 3.5 on the die-rolling task in the “added to participants” condition and “deducted from opponents” condition were classified as ‘lying’; self-reported points below 3.5 on the die-rolling task in the “added to opponent” condition were classified as ‘lying’.

**Figure 4:**
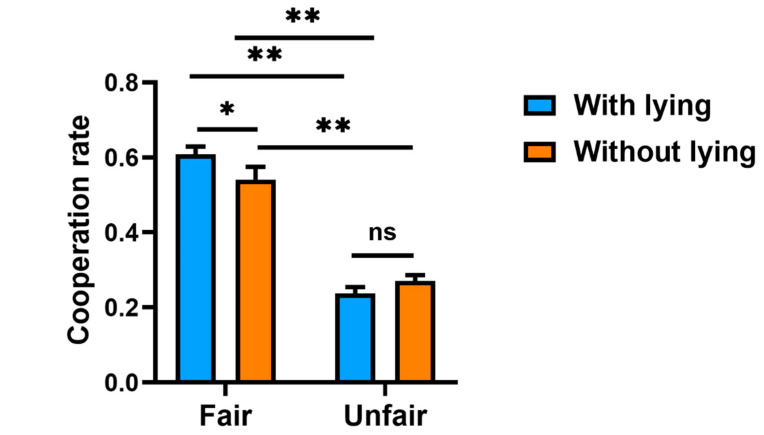
Results from the moderation analysis. The averaged cooperation rates were displayed as a function of lying (with vs. without), separately for the fair condition and the unfair condition. Error bars indicate standard error. *: *p* <0.05, ** *p* < 0.01, ns: no significant difference.

In the first analysis, cooperation rates were subjected to a 2 (fairness condition: fair vs. unfair) × 2 (lying: with vs. without) between-subjects ANOVA. There was a significant main effect of fairness, *F*(1, 236) = 214.24, *p* < 0.001, *η_p_*² = 0.48, 95% CI = [0.97, 0.98]. There was no significant main effect of lying, *F*(1, 236) = 0.63, *p* = 0.43. Importantly, there was a significant interaction between fairness and lying, *F*(1, 236) = 5.10, *p* = 0.025, *η_p_*² = 0.02. Simple effect tests (Figure 4 showed significant differences in cooperation rates between lying and not lying in the fair treatment condition (*F*(1, 236) = 4.48, *p* < 0.05, *η_p_*² = 0.02, 95% CI = [0.001, 0.07]) and no significant effect of unfairness on lying [*F*(1, 236) = 1.12, *p* > 0.05]. Specifically, there was a significant effect of not lying on fairness condition (*F*(1, 236) = 67.71, *p* < 0.001, *η_p_*² = 0.22, 95% CI = [0.14, 0.31]); participants without lying cooperated more in the fair condition (*M* = 0.54, *SD* = 0.22) than in the unfair condition (*M* = 0.27, *SD* = 0.13). There was a significant effect of lying on fairness condition (*F*(1, 236) = 164.35, *p* < 0.001, *η_p_²* = 0.41, 95% CI = [0.32, 0.49]). Participants with lying cooperated more in the fair condition (*M* = 0.61, *SD* = 0.18) than in the unfair condition (*M* = 0.24, *SD* = 0.12).

In the second moderation analysis, the distance of the average number of points reported by the participants from the expected value (3.5) served as a measure of lying. Specifically, reported average die-rolling points greater than 3.5 were considered as self-serving lying in cases where points were added to participants’ accounts or deducted from opponents’ accounts. Reported average die-rolling points less than 3.5 were considered as self-serving lying in cases were the obtained points were added to the opponent’s account. Here, we tested with a regression model the perceived fairness of the opponent, an index of lying (absolute value self-reported points minus 3.5), and their interaction influence cooperation (*n* = 240). Perceived fairness had a significant positive effect on cooperation (*β* = 0.06, *t* = 6.63, *p* < 0.001, 95% CI = [0.04, 0.08]), the index of lying had a significant negative effect on cooperation (*β* = −0.05, *t* = −4.49, *p* < 0.001, 95% CI = [−0.07, −0.03]), and the interaction had a significant negative effect on cooperation (*β* = −0.02, *t* = −2.61, *p* < 0.05, 95% CI = [−0.03, −0.04]) [58].

## 4. General Discussion

In previous studies on fairness’ effect on cooperation or lying, the findings were always isolated. We conducted two experiment to test the relation between fairness, cooperation, and lying. The findings of two studies indicate that (a) unfairness can reduce people’s subsequent cooperation (Experiment 1 and 2) and increase self-serving dishonesty (Experiment 2); (b) dishonesty reduces cooperation but also weakens the effect of fairness treatment on cooperation (moderation analysis); and (c) when people are treated fairly and employ self-serving lies, their cooperation increases. Specifically, people primed with lower perceived fairness in the condition (the partner defected in 80% of trials) had less cooperation and more dishonesty. Moreover, participants who were in the fair condition and lied cooperated more compared to people in the other conditions.

First, the results of this study supported fairness norm. Inspired by a large volume of experimental evidence, there has been much work on social (as opposed to individual) preferences, reflecting individuals’ concern for fairness in the income distribution [24,59]. The results of this study also supported the inequity aversion theory. One key pillar of fairness perception is equality, or the social norm that people deserve a similar share of the pie if no effort is involved [60]. Fairness is explicit or implicit rules that define context-specific appropriate behavior [30]. When this principle is violated, people seek ways to restore a sense of fairness [61,62,63]. Substantiating this view, and in line with prior research [64,65], we demonstrate that cooperation rates (Experiment. 1 and 2) are reduced when players experience unfairness. In line with social exchange theories, people try to restore justice through reciprocation and through emphasizing their own benefit rather than the global benefit [27,28]. This provides support for the claim that egalitarianism embeds important motives that might underlie cooperation [66]. Our findings also indicate that when people perceive that their counterparts are insufficiently cooperative, they perceive the situation as unfair (Experiment 2). These findings are aligned with evidence from behavioral economics, suggesting that perceptions of fairness are influenced by the intentions of the interaction partner [67] and that humans are less cooperative when encountering unfairness [68].

Second, the findings provide support for the claim that perceived unfairness can promote dishonest behaviors, as manifested in self-serving reports of die-rolling trials [32]. Specifically, when facing unfair treatment in the prisoner’s dilemma game and the die-roll outcome is either added to self or deducted from the opponent’s account, participants report upward biased points, but when the outcome is added to the opponent, they report downward biased points. In contrast, when the outcome is added to the opponent’s account, participants report downward biased points (Experiment 2). This is provides a more granular view of the principle observed in prior research: that fairness violations can result in moral disengagement [40], motivate fairness restoration through dishonest and even illegal behavior [41], and ultimately, increases dishonesty and corruption [69]. For instance, employees who are treated unfairly present increases in employee theft [41], and negative actions of counterparts in a bargaining game increase dishonest behavior [70]. Overall, we show that materialistic self-interest is not the only motivational force for dishonesty, because other factors such as social fairness also influence the utility functions of economic agents [71]. We demonstrate here that this principle affects behaviors in various forms of point allocation that reflect either more points than deserved to oneself and/or less points than deserved for others. This is an important extension of prior research that has primarily focused on cheating for personal gains, because inflicting suffering on others can also be rewarding, represent justice restoration, and motivate behaviors [72,73].

Importantly, these findings illuminate the important interplay between fairness treatment/perceptions and self-serving dishonest behaviors in promoting (or inhibiting) cooperation. This integrative perspective extends prior research, which has primarily focused independently on dishonesty or fairness as standalone drivers of cooperation. As we show here, fair treatment and dishonesty can serve as inter-related mechanisms that independently, and together, through substitutional relationships, can motivate cooperation. This extension is important because we live in a social context that frequently requires interactions and cooperation with others, including with unrelated strangers [8,74] (inequity aversion theory).

Our study provides an integrative perspective that accounts for simultaneous dishonest behavior and unfair treatment effects. It is unreasonable to isolate them; as prior studies [70] have shown, and as we also show here, unfair treatment promotes dishonest behaviors. We further demonstrate that these two factors are not only associated, but also interact to influence cooperation. This is in line with the notion that people often cheat when proper incentives are in place, but not to the full extent possible [32,52,75]. They instead regulate their cheating behaviors, in part to avoid unpleasant feelings of guilt, shame, and regret [76,77,78,79]. Another possible way to avoid such unpleasant feelings, especially if cheating has already been conducted, is through compensatory actions that will mitigate such feelings. In our case, it is possible that cooperation is such a behavior that can help people compensate for the expected adverse emotional effects of cheating. It can also nullify fairness (unfairness) effects, because it reduces the need for defection as a means to restore equity. Simply put, because dishonest behavior can restore equity [42]; it reduces the motivation to defect, as people may aim to get just the right level of equity, and try to avoid going beyond the needed reward. This is line with the notion that expectation of such unpleasant moral emotions can also drive corrective and preventative actions [44,45,46,47]. Here, we show that such actions can include increased cooperation, as presented by people who were treated fairly, but still decided to lie.

From a practical standpoint, the findings can explain inconsistent human behaviors across contexts. For example, they can explain why politicians can lie to get what they want, and then cooperate with the competing party. This mechanism can be used by individuals, firms, and nations for promoting cooperative behavior. If such entities can drive the counterpart to cheat (e.g., by creating cheating opportunities and motivations, like those created by the die rolling task), while the counterpart is treated fairly, they can expect, based on our findings, to have increased cooperation from the counterpart.

### Limitations

While this study is unique in combining fairness, dishonest behavior, and cooperation, several limitations should be acknowledged. First, we used one mechanism to instigate unfairness perceptions (defection) in one task (prisoner’s dilemma game), and measured fairness perception rather subjectively and simplistically. Future research can test our assertions with other tasks, using different manipulations of fairness, and supplementing the subjective measures of fairness with bio-physiological responses, such as activation of the DLPFC and insular cortex [2,80]. Second, we assumed that moral emotions such as guilt and shamed propelled higher cooperation when cheating in the fair treatment condition, but we did not capture them. Future research can extend our model in this direction. Lastly, future research can fine tune our findings by accounting for some stable various relevant dispositions, personality traits (e.g., competitiveness, Machiavellianisms, and social value orientation), and gender.

## 5. Conclusions

To conclude, fairness is an important driver of both cooperation and honesty. More perceived fairness arouses cooperation and less dishonesty. The present research also shows that it not only influences dishonest behavior but also interacts with it to influence cooperation rates. This suggested that lying/dishonesty acted as a direct inhibitor of cooperation and as a significant moderator that reduced the effect of perceived fairness on cooperation. In the fair condition, lied more, cooperated more. These findings pave the way for important future research, and suggests, from a practical standpoint, that occasional dishonest behaviors can reduce cooperation but also marginalize the effect of fairness on cooperation.

## Figures and Tables

**Table 1:** The payoff matrix of the game.

		The Other Player	
		Cooperate	Defect
Participant	Cooperate	(2, 2)	(3, −1)
	Defect	(−1, 3)	(1, 1)

## Data Availability

The data presented in this study are available on request from the corresponding author.
